# Single collagen fibrils isolated from high stress and low stress tendons show differing susceptibility to enzymatic degradation by the interstitial collagenase matrix metalloproteinase-1 (MMP-1)

**DOI:** 10.1016/j.mbplus.2023.100129

**Published:** 2023-02-21

**Authors:** Kelsey Y. Gsell, Samuel P. Veres, Laurent Kreplak

**Affiliations:** a School of Biomedical Engineering, Dalhousie University, Halifax, Nova Scotia, Canada; b Division of Engineering, Saint Mary’s University, Halifax, Nova Scotia, Canada; c Physics and Atmospheric Science, Dalhousie University, Halifax, Nova Scotia, Canada

**Keywords:** Collagen fibril, MMP-1, Collagenase, AFM, Tendon

## Abstract

Bovine forelimb flexor and extensor tendons serve as a model for examining high stress, energy storing and low stress, positional tendons, respectively. Previous research has shown structural differences between the collagen fibrils of these tissues. The nanoscale collagen fibrils of flexor tendons are smaller in size, more heavily crosslinked, and respond differently to mechanical loading. Meanwhile, energy storing tendons undergo less collagen turnover compared to positional tendons and are more commonly injured. These observations raise the question of whether collagen fibril structure influences the collagen degradation processes necessary for remodelling. Atomic force microscopy was used to image dry collagen fibrils before and after 5-hour exposure to matrix metalloproteinase-1 (MMP-1) to detect changes in fibril size. Collagen fibrils from three tissue types were studied: bovine superficial digital flexor tendons, matched-pair bovine lateral digital extensor tendons, and rat tail tendons. Compared to control fibrils exposed only to buffer, a significant decrease in fibril cross-sectional area (CSA) following MMP-1 exposure was observed for bovine extensor and rat tail fibrils, with larger fibrils experiencing a greater magnitude of CSA decrease in both fibril types. Fibrils from bovine flexor tendons, on the other hand, showed no decrease in CSA when exposed to MMP-1. The result did not appear to be linked to the small size of flexor fibrils, as equivalently sized extensor fibrils were readily degraded by the enzyme. Increased proteolytic resistance of collagen fibrils from high stress tendons may help to explain the longevity of collagen within these tissues *in vivo*.

## Highlights

• Collagen fibril enzymatic degradation was measured using atomic force microscopy.
• Fibrils from low-stress positional tendons decreased in size on exposure to MMP-1.
• Fibrils from paired, high-stress energy storing tendons showed no change in size.
• For positional tendons, amount of degradation was dependant on initial fibril size.
• Tendon collagen turnover differences may be related to MMP-1 susceptibility of fibrils.

## Introduction

Collagen is the most abundant protein in the body of humans and almost all other animals. While there are variations in the molecular and supramolecular structure of collagen, type I collagen predominates, taking the form of nanoscale rope-like supramolecular assemblies called collagen fibrils. Collagen fibrils act as the primary structural units and tensile elements of load-bearing connective tissues. Owing to the reciprocal relationship between structure and function, collagen fibril structure varies between tissues [1], [2], within a specific tissue type [3], [4], and even within a single tissue [5].

Tendon is an ideal tissue for the study of collagen structure–function because it is made predominantly of well-aligned, type I collagen fibrils. The general function of tendons is to transfer tensile force between muscle and bone. However, this function can be specialized, and a popular model that is used to study collagen structure and function are the opposing tendons on the forelimb of large quadruped animals [3], [4], [6], [7], [8], [9], [10], [11]. The dorsal extensor tendons are classified based on their function as positional tendons, while the palmar superficial digital flexor tendon is an energy storing tendon similar to the human Achilles [3], [12]. Positional tendons mainly function for fine control of movement and positioning, while energy storing tendons are spring-like tendons that experience much higher stresses and strains, and store strain energy that can be returned to the skeleton to aid in movement [3], [12].

Structurally, energy storing tendons are composed of smaller diameter fibrils [4], [13] and have increased lateral connections both between and within collagen fibrils by way of associated molecules/proteins and intermolecular crosslinks, respectively [3], [4], [10], [11]. Functionally, intermolecular crosslinks limit the sliding of adjacent molecules which results in distinctly different loading curves between individual collagen fibrils from these functionally distinct tendons, and consequently, unique modes of failure due to tensile overload [14], [15], [16], [17]. The greater plasticity at high stress seen in the fibrils of positional tendons may be linked to the higher tensile strength of these tissues, while under cyclic loading the reduced plasticity of energy storing fibrils may help to limit accumulation of fatigue damage [4].

In addition to impacting mechanics, differences in collagen crosslinking between functionally distinct tissues may have another consequence. Crosslinking of collagen molecules within a fibril may limit the ability for enzymes such as the primary collagenases, matrix metalloproteinases (MMPs) 1, 8, and 13, to bind, unwind, and cleave the molecules. It is possible that the increased crosslinking of collagen fibrils from energy storing tendons may inhibit cleavage by the enzymes required for maintenance and/or remodelling [18], potentially also slowing healing processes in these commonly injured tissues [19], [20]. Indeed, the collagen of energy storing tendons shows significantly slower turnover compared to that in positional tendons [3], [11], despite greater cellularity [3], [8], [9].

The study of individual collagen fibrils is difficult due to their nanoscale cross-section. Many imaging modalities require fixation or destructive preparation to resolve features at this scale. Atomic force microscopy (AFM) is a unique tool because it allows for imaging in both ambient and wet conditions and does not destroy the sample, allowing for longitudinal and interventional study. As the primary structural unit of connective tissues throughout the body, studying collagen fibrils is of great relevance, not only to understanding native tissues in health and disease, but also to effectively harness collagen protein for biomaterial and biomedical applications.

In the present work we have isolated individual collagen fibrils from functionally distinct tendons and compared their enzymatic susceptibility to the interstitial collagenase MMP-1. This was achieved by imaging collagen fibrils with AFM before and after exposure to MMP-1 and determining changes in fibril size. We demonstrate that there are differences in fibril degradation depending on both fibril size and tissue of origin (tendon type).

## Results

### Collagen fibrils from high stress tendons are more resistant to enzymolysis by MMP-1

**Fig. 1:**
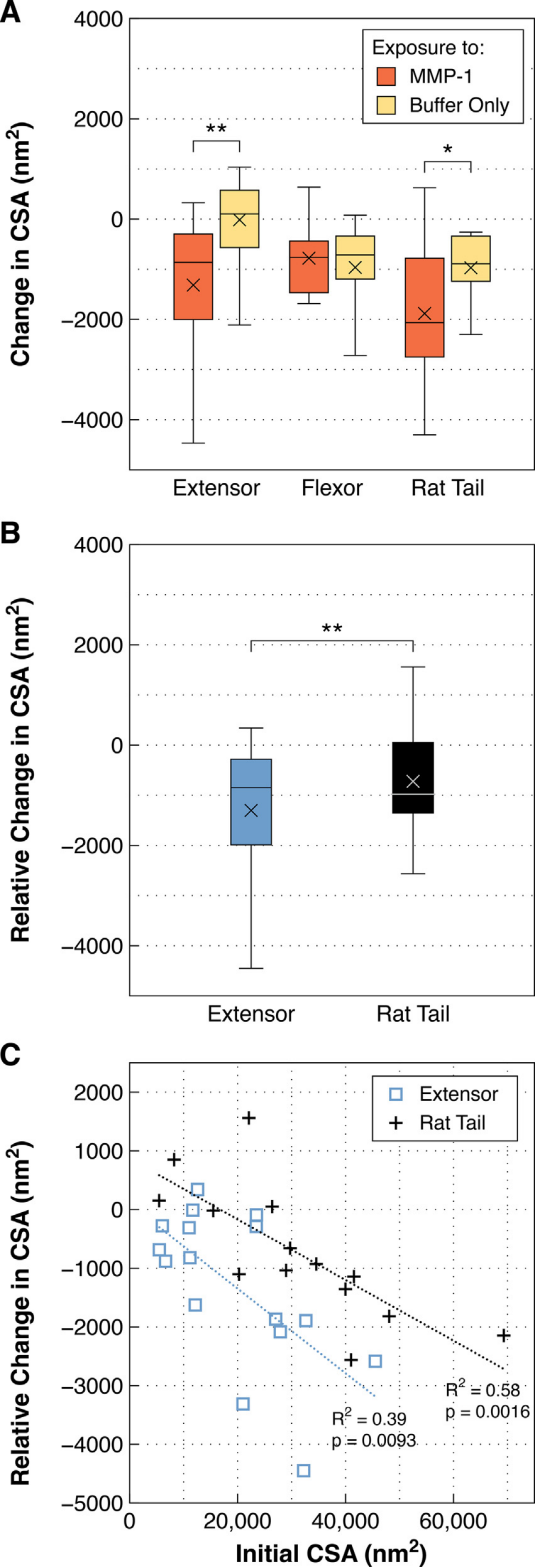
**A)** Change in cross-sectional area (CSA) of single collagen fibrils exposed to MMP-1 in buffer or buffer alone. Fibrils from bovine digital extensor tendons and rat tail tendons exposed to MMP-1 experienced significant decreases in CSA compared to fibrils from the same tendons exposed to buffer only. In contrast, the CSA of fibrils from high stress bovine superficial digital flexor tendons remained unchanged following exposure to MMP-1. **B)** Fibrils from bovine extensor tendons experienced greater reduction in CSA than those from rat tail tendons. **C)** For collagen fibrils from both bovine digital extensor and rat tail tendons, greater reductions in CSA following MMP-1 exposure were seen for fibrils of larger initial CSA. **p < 0.01, *p < 0.05.

The ability of MMP-1 to remove material from collagen fibrils varied by tendon type. Collagen fibrils from both bovine lateral digital extensor tendons and rat tail tendons experienced significant decreases in cross-sectional area following exposure to MMP-1 (*p* = 0.0025 and 0.0481, respectively), while no change was observed for fibrils from bovine superficial digital flexor tendons (Fig. 1A). Taking into account CSA change for fibrils in the control group, the relative CSA decrease observed following MMP-1 exposure for fibrils from extensor tendons was significantly greater than that observed for rat tail tendon fibrils (*p* = 0.0022; Fig. 1B).

For both bovine extensor and rat tail tendon fibrils, an effect of initial fibril size on degradation by MMP-1 was observed. Significant linear relationships existed between initial CSA and relative change in CSA following enzyme treatment, where larger fibrils experienced greater degradation (Fig. 1C).

**Fig. 2:**
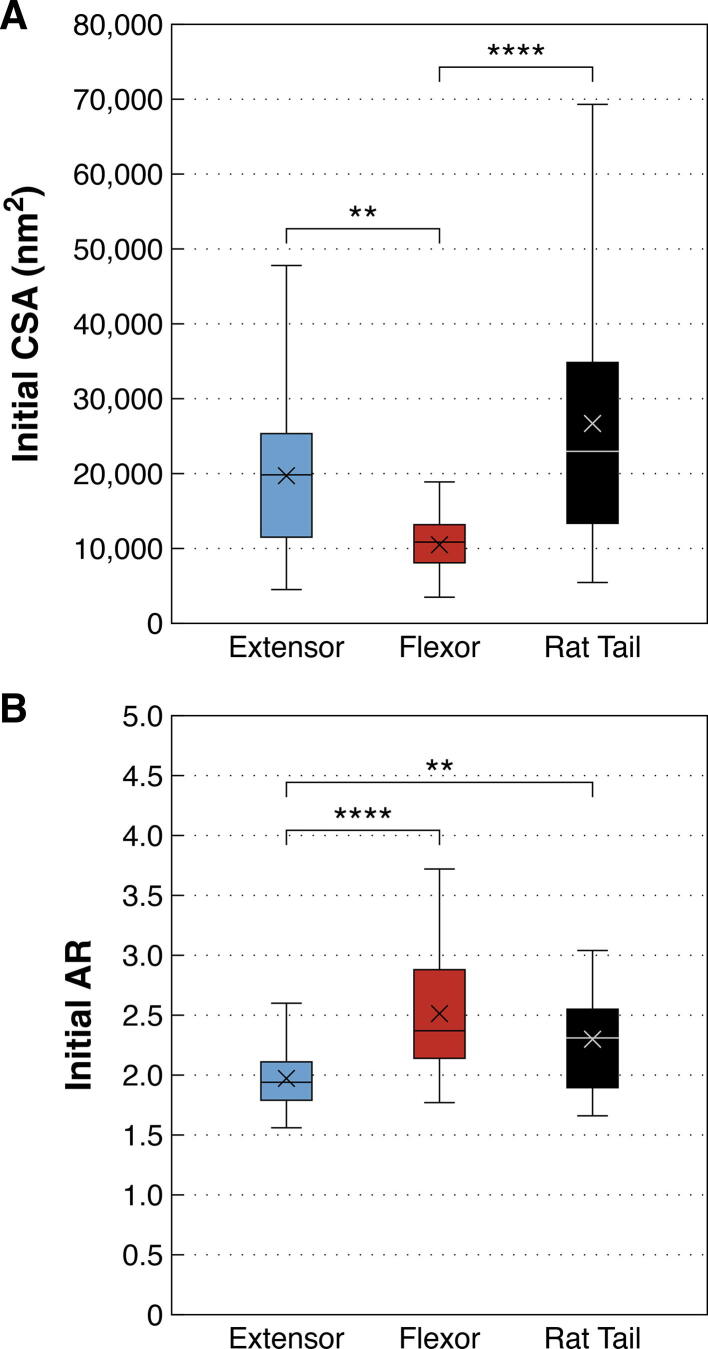
Cross-sectional area (CSA) and aspect ratio (AR) of individual collagen fibrils isolated from bovine lateral digital extensor, bovine superficial digital flexor, and rat tail tendons prior to incubation (control and enzyme groups pooled). **A)** Bovine flexor fibrils were significantly smaller than extensor and rat tail fibrils. **B)** Bovine extensor fibrils were significantly less flattened than flexor and rat tail fibrils. **** *p* < 0.0001, ** *p* < 0.01.

**Fig. 3:**
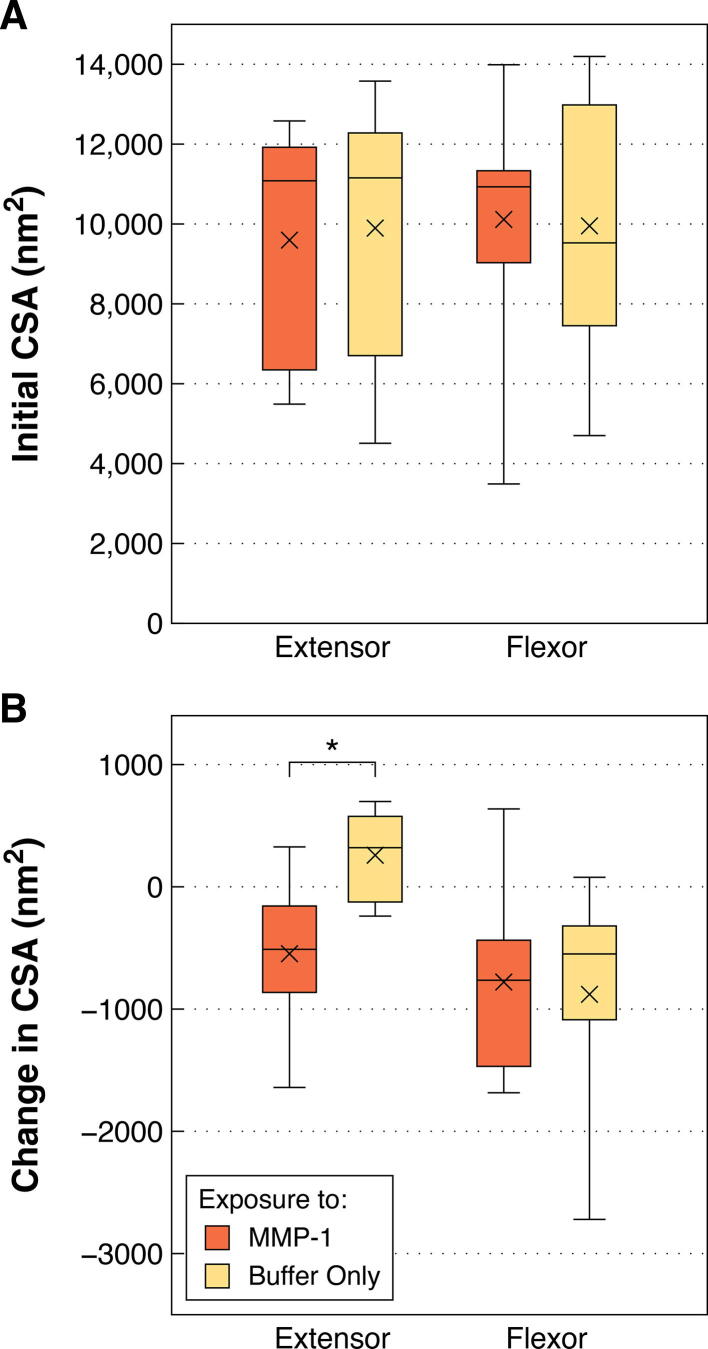
**A)** Bovine flexor fibrils were compared to a subset of similarly sized bovine extensor fibrils (initial CSA < 15,000 nm^2^). There were insufficient rat tail fibrils of this size to include in the comparison. **B)** Unlike flexor fibrils, the size-matched extensor fibrils showed significant CSA loss following MMP-1 exposure, suggesting that the lack of degradation seen in the flexor fibrils was not due to their small size. * *p* < 0.05.

The effect of initial fibril size on degradation by MMP-1 was explored in relation to the lack of CSA change seen for bovine flexor fibrils. As expected from previous work [4], [21], [22], bovine flexor fibrils were significantly smaller than both bovine extensor and rat tail fibrils (*p* = 0.0013 and < 0.0001, respectively; Fig. 2A). To assess the effect of small initial fibril diameter, the response of the bovine flexor fibrils was compared to an equivalently sized subsample of the bovine extensor fibrils (those with initial CSA < 15,000 nm^2^; Fig. 3A). Unlike flexor fibrils, the similarly sized extensor fibrils still showed a significant decrease in CSA following enzyme exposure (*p* = 0.0155; Fig. 3B), indicating that small size alone was not the cause of the flexor fibrils’ resistance to degradation by MMP-1.

### Fibril shape change following MMP-1 exposure differs for fibrils from high stress tendons

The dry cross-sectional shape of individual collagen fibrils adhered to glass varied between tendon types following sample preparation. While all fibrils assumed flattened ellipsoid shapes, as indicated by aspect ratios greater than 1, bovine extensor fibrils were significantly less flattened compared to both flexor (*p* < 0.0001) and rat tail fibrils (*p* = 0.0042; Fig. 2B).

**Fig. 4:**
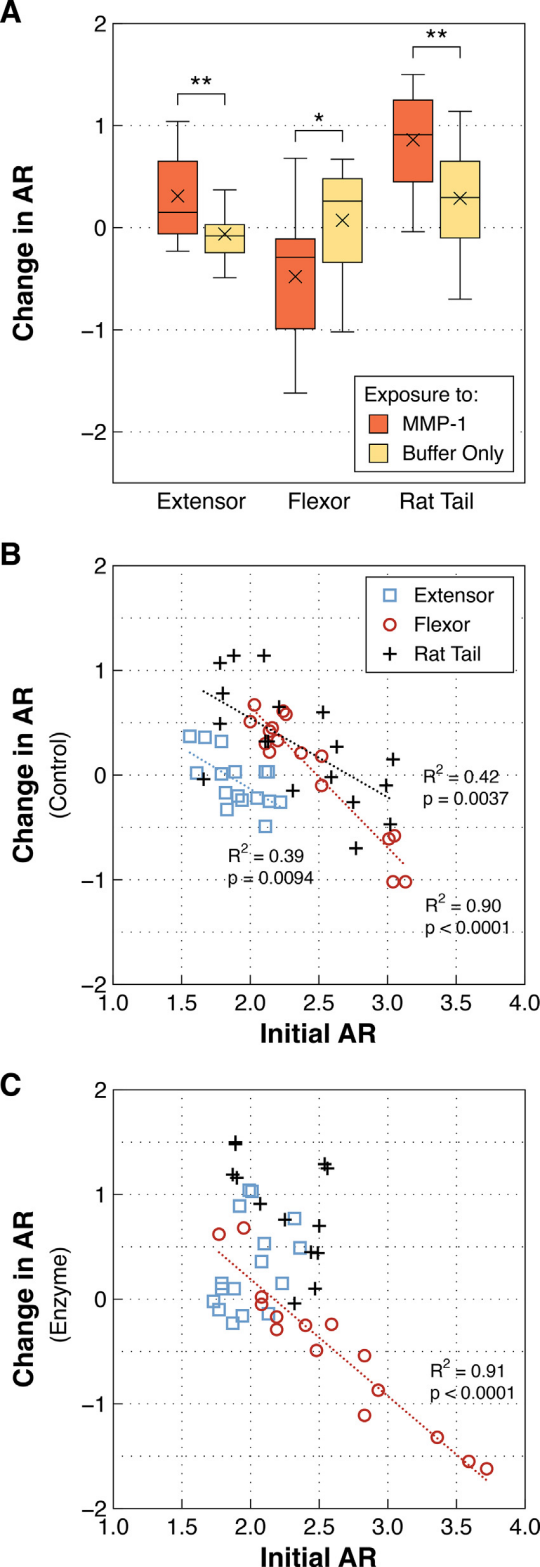
**A)** Change in aspect ratio (AR) of single collagen fibrils exposed to MMP-1 in buffer (enzyme group) or buffer alone (control group). While exposure to MMP-1 led to flattening (increased AR) of both bovine extensor and rat tail fibrils, fibrils from bovine flexor tendons were raised following enzyme treatment. **B)** After exposure to buffer only, fibrils that were initially flatter or more raised tended to become less flat and less raised, respectively. This was true for all three fibril types. **C)** The relationship between initial AR and change in AR seen for the control fibrils was preserved for bovine flexor fibrils following MMP-1 exposure, but not for bovine extensor or rat tail fibrils. **p < 0.01, *p < 0.05.

Following treatment and subsequent dehydration, the dry aspect ratio (AR, described in Section 5.4) of collagen fibrils changed more when exposed to MMP-1 than compared to buffer alone. For extensor and rat tail fibrils, exposure to MMP-1 resulted in significant flattening of the fibrils marked by increases in AR (*p* = 0.0067 and 0.0058, respectively; Fig. 4A). Flexor fibrils, on the other hand, reacted oppositely, showing significantly raised geometries and corresponding reductions in AR (*p* = 0.0362; Fig. 4A). Interestingly, for the control study, all three fibril types showed similar linear relationships between initial AR and change in AR. Individual fibrils with more flattened geometries initially were less flattened following exposure to the buffer solution, and vice versa, with the magnitude of change corresponding to how far the initial AR was from the average for that tendon type. In other words, very flat fibrils experienced more ‘raising’ than less flat fibrils (compared to the average value), and vice versa (Fig. 4B). Following exposure to MMP-1, this relationship was maintained only for the bovine flexor fibrils, while being lost for both the extensor and rat tail fibrils (Fig. 4C).

### Hydration effects on fibril shape and size

**Fig. 5:**
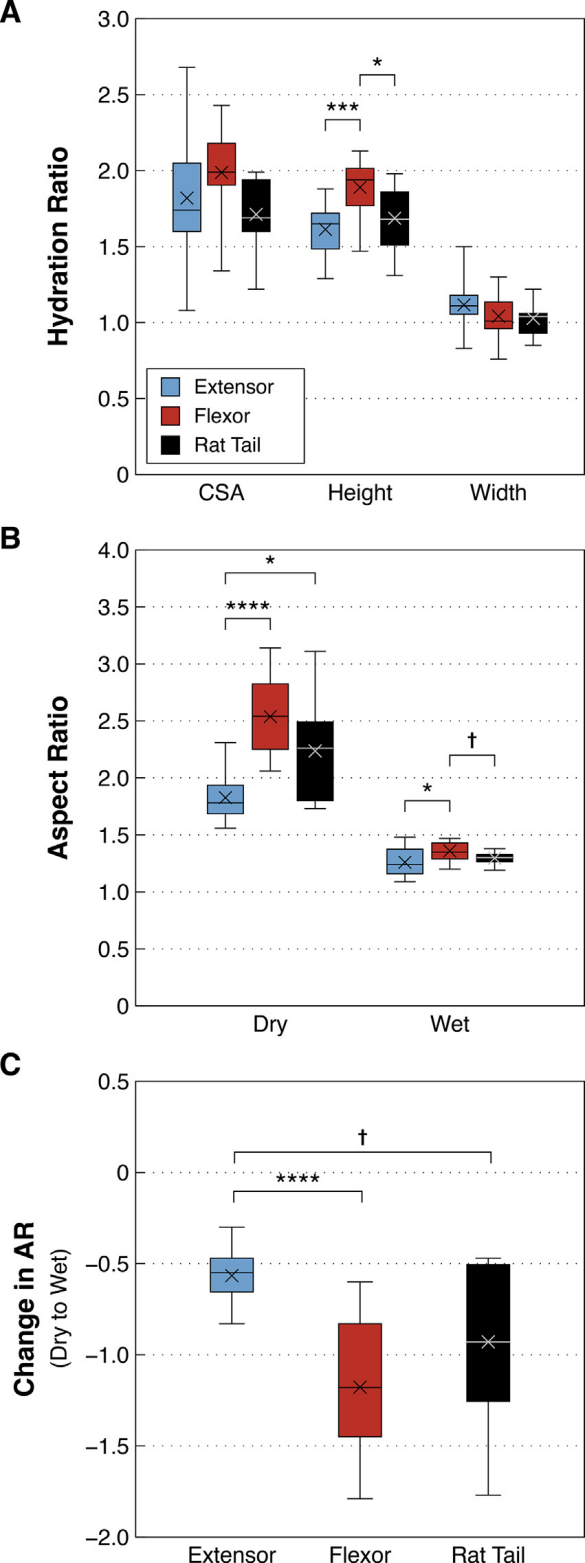
Characterization of the swelling behaviour of individual collagen fibrils when hydrated. **A)** Hydration ratios indicate that fibrils from bovine flexor tendons experienced larger swelling in the height dimension compared to bovine extensor and rat tail fibrils. All three fibril types shared similar hydration ratios in width and CSA. **B)** Differences in fibril aspect ratio (AR) in the dry compared to wet state. **C)** The change in AR moving from dry to wet state for the three fibril types studied. ****p ≤ 0.0001, ***p < 0.001, *p < 0.05, †*p*≈0.07.

To understand whether the different fibril types used would have had differing aspect ratios during incubation with MMP-1, a hydration study was conducted. From the dry state, when hydrated in buffer fibrils from all three tendons experienced similar CSA swelling (Fig. 5A). However, the swelling of hydrated fibrils was anisotropic: swelling occurred primarily in the height dimension, with little change in width (Fig. S2), the latter indicated by hydration ratios close to 1 (Fig. 5A). While CSA and width hydration ratios were similar between fibril types, hydration ratio for height differed, with flexor fibrils swelling in height significantly more than both extensor (*p* = 0.0009) and rat tail (*p* = 0.0152) fibrils (Fig. 5A).

Anisotropic swelling was also indicated by the shape changes experienced by fibrils upon hydration. When hydrated, all fibrils experienced decreased ARs, signifying an increase in height relative to width. While differences in aspect ratio between fibril types were much reduced in the hydrated state, bovine flexor fibrils were slightly flatter in the wet state (larger AR) than bovine extensor fibrils (*p* = 0.0494), and, though not significantly, rat tail fibrils (*p* = 0.0697; Fig. 5B). Change in AR moving from dry to hydrated state was smallest for bovine extensor fibrils and largest for bovine flexor fibrils, with rat tail fibrils being statistically similar to both (Fig. 5C).

## Discussion

The collagen fibrils of high stress, energy storing tendons are known to be differently structured than those of low stress, positional tendons, with functional consequences in terms of mechanical response and mechanical damage resistance [4], [6], [7], [17]. In the present study, collagen fibrils from bovine lateral digital extensor tendons and rat tail tendons—low stress, positional tendons—showed evidence of degradation by the collagenase MMP-1 over 5 hrs of exposure. Conversely, fibrils from bovine superficial digital flexor tendons—high stress, energy storing tendons—were not degraded. These findings indicate that the collagen fibrils of high stress, energy storing tendons may possess greater resistance to enzymatic cleavage than those of low stress, positional tendons. It is intriguing that the difference in MMP-1 susceptibility seen between fibrils from energy storing and positional tendons mirrors the *in vivo* variation in collagen molecule longevity in these two tissues [11]. Could the slow collagen turnover seen in high stress tendons [11], [23], [24] be caused, at least in part, by a high resistance of their collagen fibrils to enzymatic removal? The results of the current work suggest that this may be possible.

### Why were collagen fibrils from bovine superficial digital flexor tendons not degraded by MMP-1?

Fibril size, measured as initial, pre-treatment CSA, influenced the amount of degradation that was seen for extensor and rat tail fibrils. Though flexor fibrils were smaller than extensor or rat tail fibrils, the smaller size of flexor fibrils does not appear to have been the basis for their resistance to MMP-1 degradation, as similarly sized extensor fibrils showed significant evidence of degradation.

There are known structural differences between bovine flexor and extensor fibrils that may be responsible for limiting degradation of the flexor fibrils by MMP-1. Previous research in the same bovine forelimb model revealed that collagen molecules in flexor fibrils are more tightly packed (reduced intermolecular spacing) and more densely crosslinked than those in extensor tendons [4]. In the analogous equine forelimb model, superficial digital flexor tendons have been found to possess consistently higher levels of the mature, trivalent crosslink hydroxylysyl-pyridinoline than exist in the digital extensor tendons [3], [10], [25], consistent with differences in the thermal behaviour of bovine flexor and extensor tendons [4].

It is possible that the increased molecular packing and crosslinking of the flexor fibrils limits the ability of MMP-1 to bind and unwind the collagen triple helix in order to cleave individual strands of the molecule [18]. This could result in either full or partial inhibition of cleavage in flexor fibrils, making detection of changes in CSA after only 5 hrs unlikely. Indeed, in unloaded tendon, increased crosslinking, albeit induced advanced glycation crosslinking rather than enzymatic crosslinking, has been found to limit degradation by bacterial collagenase [26]. It is also possible that cleavage of superficial molecules on flexor fibrils did indeed occur, but that their fragments were retained in place due to the higher level of intermolecular crosslinking present, shielding underlying molecules from cleavage. The situation *in vivo* may differ, where for instance gelatinases such as MMP-2 and -9 could aid in the removal of these cleaved fragments.

In addition to structural differences between the collagen fibrils themselves, differences in proteoglycan association with the three types of fibrils used in the present study could also have impacted degradation by MMP-1. In a study of *in vitro* assembled atelo-collagen molecules (molecules lacking the non-helical telopeptides and thus any crosslinking), incubation with decorin, fibromodulin, and lumican each had an inhibitory effect on subsequent degradation by MMP-1, with decorin yielding the most inhibition [27]. These are the main proteoglycans found in tendon along with biglycan, and are small leucine rich proteoglycans (SLRPs) with a core protein 40–60 kDa in size that binds to collagen molecules within the gap regions of fibrils. Among equine forelimb tendons, the flexor tendon was found to have increased mRNA expression of all 4 SLRPs [25], consistent with measurements of greater glycosaminoglycan levels in these tendons compared to extensors [3], [8], [25]. While some of this difference may be attributable to variation in the interfascicular matrix content and composition between these two tendons, particularly in regard to lubricin content [28], the flexor fibrils used in the present study may well have had greater surface coverage by proteoglycans than either the extensor or rat tail fibrils. This may have been particularly true for decorin, which is known to limit fibril size and is the dominant proteoglycan in tendon [29]. Recent AFM measurements of surface charge density along fibrils provides some support that differences in bound proteoglycan coverage may have existed between the fibrils used. Using fibrils prepared in the same manner as the current study, Mull & Kreplak [30] observed differences in surface charge present at the decorin binding site between bovine flexor and extensor fibrils, though were unable to confirm that this was indeed due to differences in bound proteoglycan.

### Age of fibrils may contribute to the size bias of MMP-1 activity on bovine extensor and rat tail fibrils

For both bovine extensor and rat tail fibrils, the present study found that fibrils with larger cross-sectional area experienced greater degradation by MMP-1. It is possible that natural tissue aging and/or remodelling processes within the tendons might have contributed to this effect. Studies in rat tail tendons [31] and in equine flexor tendons [10] show that as animals age, the cross-sectional size of fibrils peaks around skeletal maturity and then decreases slightly with further aging. The animals used in the current study were skeletally mature, yet young in their overall life span (∼10%). Perhaps the smaller diameter fibrils used were more recently synthesized than the larger diameter fibrils. When fibrils are newly synthesized, they are small and are more highly associated with decorin and bigylcan, which act to limit lateral growth while fibril length increases [32], [33]. It is possible that the small extensor and rat tail fibrils used possessed greater surface coverage by SLRPs, contributing to the size bias in degradation observed. Even so, such a finding may have limited relevance *in vivo*, as small diameter fibrils may be degraded through an alternate pathway compared to the more well-established fibrils typically thought to be degraded primarily by MMP-1. A recent study by Chang et al. [34] looking at the rat Achilles tendon found that over a 24 hr period, degradation of very small diameter fibrils (<75 nm) occurred via Cathepsin K, which requires the glycosaminoglycan side chains of proteoglycans to be present in order to cleave collagen [35].

While small, more recently synthesized fibrils may show greater resistance to MMP-1 cleavage, larger, more mature fibrils may be more susceptible to cleavage. With increasing age, collagen fibrils accumulate non-enzymatic advanced glycation end-product (AGE) crosslinks [36], [37]. AGEs can theoretically occur at multiple locations along the molecule rather than at the two specific head to tail sites of enzymatic crosslinks. As AGE crosslinks develop within collagen fibrils, intermolecular spacing increases [37], [38], enzymatic crosslinks decrease, and interactions with proteoglycans decrease [39]. Thus, as opposed to high levels of AGE crosslinking which may limit cleavage of unloaded fibrils by MMP-1 [26], low levels of AGE crosslinking and the corresponding increase in lateral, intermolecular spacing may increase vulnerability by easing access to the cleavage site. Indeed, increased molecular packing of collagen molecules decreases configurational entropy, thereby limiting enzyme accessibility to cleavage sites and therefore degradation [18], [40]. The present study’s observed relationship between fibril size and degradation warrants further study and validation.

### Fibril diameter selection

It is known that bovine flexor fibrils are smaller in cross-sectional dimension than those from paired bovine extensor tendons. In a prior study [4], approximately 400 fibrils each from the superficial digital flexor and common digital extensor tendons of 4 animals were measured using scanning electron microscopy. Flexor fibrils ranged in diameter from 23 to 184 nm (mean 80 nm) while extensor fibrils ranged from 37 to 237 nm (mean 134 nm). Estimating diameter from the CSA measured in the current study, dried flexor fibrils across all three treatments ranged from ∼ 67 to 155 nm in diameter (mean 115 nm), while extensor fibrils ranged from ∼ 76 to 259 nm in diameter (mean 158 nm). The initial dried fibril size of all rat tail fibrils imaged ranged from 83 to 301 nm (mean 186 nm), which is similar to reports in the literature of averages of 185 nm [21] and 210 nm [22] at 12 weeks of age, with fibrils ranging from<40 nm to over 500 nm [21]. Fibrils were identified as targets for AFM using optical microscopy prior to imaging. Due to the diffraction limit of visible light, fibrils close in size to the minimums reported by Herod et al. [4] were not visible [41], resulting in bias towards fibrils larger than the true minimums present in these tendons.

### Methodological implications on dry size and hydration ratio measurements of single fibrils

Collagen fibrils adhere to underlying substrates upon dehydration, and due to this adherence fibrils flatten and take on an elliptical cross-section. This has been reported and discussed briefly in other studies using AFM (and TEM) [41], [42], [43]. However, most AFM studies report only fibril height and equate this to fibril diameter. The flattening that occurs during dehydration may be influenced by the internal structure of the fibril or due to interactions with the substrate and/or aqueous solutions during sample preparation. For measurements taken with AFM, these shape changes may significantly influence the comparison of height between different fibrils or of the same fibril following treatment.

In the present study, following adherence to the bottom of a glass dish and subsequent dehydration, fibrils from different tendons adopted different shapes quantified through aspect ratio. For example, bovine flexor fibrils were flatter than bovine extensor fibrils. Following rehydration in MMP buffer and exposure to MMP-1, while not noticeably degraded, flexor fibrils experienced a reduction in AR becoming more raised, while extensor and rat tail fibrils saw increases in AR, becoming flatter. Additionally, the flatter starting shape of the flexor fibrils resulted in a larger hydration ratio of the flexor fibrils when looking only at the height dimension. These examples highlight that height change alone is not a reliable metric when assessing changes in collagen fibril size.

Height is also the size metric most commonly used in the calculation of hydration or swelling ratio in the literature. Hydration or swelling ratio of collagen fibrils can be used to examine the hydration effects of different solutions on fibrils [44], or of fibril structural properties such as molecular density and (non-enzymatic) crosslinking [45], [46], [47]. The results of the present study suggest that when possible, CSA should be used to quantify fibril size, including whole fibril hydration effects, due to the flattened shape of dry fibrils adhered to the underlying substrate, the variation in AR of these fibrils between different tendon types, and anisotropy of swellings upon rehydration. This will allow for more accurate comparisons to be made.

The adherence of fibrils to their underlying substrate in the current study is not expected to have influenced the degradation of fibrils. While flexor fibrils were flatter than extensor fibrils in the wet state, this difference was very small, and rat tail fibrils, which were similar in wet AR to the flexor fibrils did experience degradation while the flexor fibrils did not.

## Conclusions

This study is the first to directly examine the susceptibility of single, natively structured collagen fibrils from functionally distinct tendons to degradation by MMP-1. The ability of MMP-1 to degrade collagen fibrils depends on tendon type, with the fibrils of high stress, energy storing tendons showing greater resistance to degradation than those from low stress, positional tendons. The structural differences between fibril types that underpin differences in MMP-1 susceptibility may be of great significance for tissue homeostasis, remodelling, and recovery from injury, as well as application to bioengineering of collagen substrates.

## Materials and methods

### Sample preparation

**Fig. 6:**
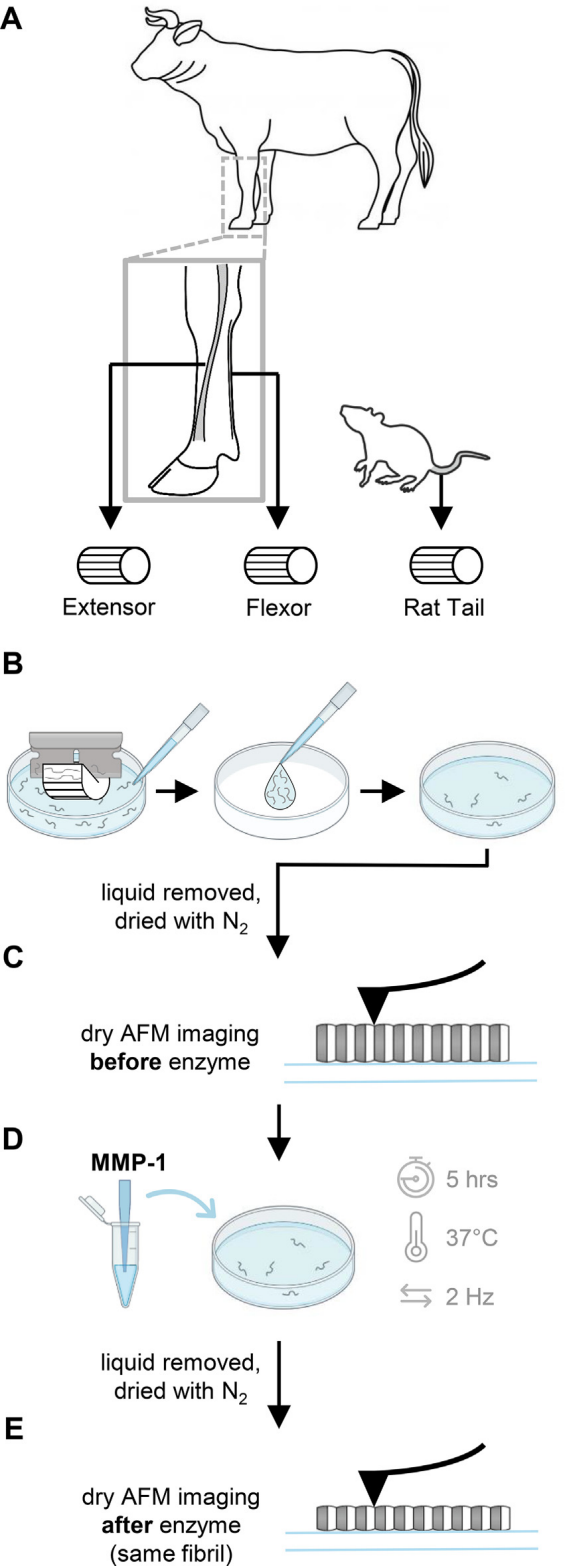
Illustration of experimental protocol for enzyme study. **A)** Tendons from the forelimb of steers and rat tails were harvested. **B)** Collagen fibrils were mechanically extracted in PBS, transferred to a glass bottom dish to adhere, and dried with N_2_ gas. **C)** Dry fibrils were imaged with atomic force microscopy (AFM). **D)** Fibrils were rehydrated in MMP buffer then incubated in buffer containing MMP-1. The reaction was quenched using EDTA (not shown). **E)** Fibrils were again dried with N_2_ gas and the same segment of each fibril was imaged with AFM.

Three tendon types were examined: paired bovine flexor and extensor tendons (the superficial digital flexor (SDF) and lateral digital extensor (LDE) tendons from the same bovine forelimb) and rat tail tendons (RTTs) (Fig. 6A). Nine tendons were collected: the paired SDF and LDE tendons of one forelimb from each of 3 adult steers (2–3 years of age), and one tail tendon from each of 3 adult male rats (2.5–3 months of age). The bovine forelimbs were collected fresh from a local abattoir, while rat tails were sourced from control animals used in other research studies at Dalhousie University. Tissue harvest was approved by the University Committee on Laboratory Animals at Dalhousie University.

To prepare samples for AFM imaging, individual fibrils were isolated from each tendon through dissection in phosphate buffered saline (PBS) (Fig. 6B) [48]. A razor blade was used to remove the outer portion of the tendon section including epitenon. The remaining tendon section was then cut into longitudinal strips and scraped using a glass rod and tweezers to release collagen fibrils into the solution. The fibril containing solution was transferred to a glass-bottom dish and left to sit for a minimum of 20 min. This allowed the fibrils to sink and adhere to the glass surface. The sample was then rinsed 3 times with ultrapure water to remove any unbound material and salt, dried using nitrogen gas, and imaged using AFM the following day. Each dish contained fibrils from one tendon and is referred to as a sample.

### Experimental protocol and AFM imaging

Sample preparation and testing was done in 3 trials, with each trial including one sample from each tendon type (samples from a pair of SDF and LDE tendons, along with a sample from one rat tail tendon). AFM measurements of individual collagen fibrils were taken before and after exposure to human recombinant MMP-1 (SRP3117, Sigma Aldrich, USA). Imaging was performed with a BioScope Catalyst AFM (Bruker, USA) using a ScanAsyst-Fluid+ cantilever (Bruker, USA) with a typical spring constant of 1 N/m and resonance frequency of 150 kHz. The spring constant was calibrated by fitting the thermal vibration spectrum of the cantilever [49]. Collagen fibrils were imaged in peak force quantitative nanomechanical mapping (PF-QNM) mode with a peak force setpoint of 10 nN, a scanning rate of 0.5 Hz, and a cantilever oscillation frequency of 1 kHz. A minimum of 5 fibrils per sample were imaged in ambient (dry) conditions at a scan size of 4 μm with 7.8 nm resolution. Following dry pre-enzyme imaging, the sample was hydrated using an MMP buffer (100 mM Tris-HCl, 10 mM CaCl2, 100 mM NaCl, and 2 uM Zn Acetate [Zn(CH_3_CO_2_)_2_], pH 7.5) [50], [51], and placed into an incubator at 37°C. After 30 min, the buffer was removed and replaced with MMP buffer containing MMP-1 (1.1 μg/ml). The sample was oscillated at 2 Hz for 5 hrs while inside the incubator. After 5 hrs of incubation with MMP-1, the enzyme solution was removed and quickly replaced with MMP buffer containing 1 mM ethylenediaminetetraacetic acid (EDTA) to quench any remaining MMP-1. After approximately 15 min the sample was rinsed and dried as before. The following day the same 4 μm length of each fibril imaged before enzyme exposure was again imaged to allow direct assessment of the structural effect of MMP-1 on the fibril. (A simplified visual depiction of these steps can be seen in Fig. 6C-E.).

In addition to the enzyme study described above, control and hydration studies were also completed with equivalent number of samples made from the same tendons as the enzyme trials. For the control study, the entire experiment was identical to the enzyme study, but with the 5 hr incubation occurring in MMP buffer only (no MMP-1). This was done to ensure that size changes reported following MMP-1 exposure were due to the enzyme alone. The hydration study was conducted to determine whether differences in swelling characteristics on hydration existed between the different fibril types. For this, 4-μm-long sections of fibrils were imaged dry, followed by hydration in MMP buffer for a minimum of 30 min, followed by hydrated imaging within the same 4 μm section. AFM imaging conditions were the same as mentioned above.

For each of the three studies, the total number of fibrils that were included in the analyses for each fibril type and condition, as well as the ambient conditions that they were imaged under, is provided in Table 1. Although reported, the variation in ambient conditions noted is not expected to have influenced outcome measures, as small variation in relative humidity causes negligible, reversible changes in fibril height of 0.04 nm/% relative humidity [52].

**Table 1:** A total of 141 fibrils from three different tendon types were examined in three different studies. Each fibril was assessed by AFM, both before (pre) and following (post) treatment. The treatments were MMP-1 exposure for the Enzyme study, and MMP Buffer exposure only for the Control and Hydration studies.

Treatment	Tendon	Number of Fibrils	Pre Temperature (°C)	Pre Relative Humidity (%)	Post Temperature (°C)	Post Relative Humidity (%)
Enzyme	LDE	16	22.2 (1.0)	25.3 (12.7)	22.9 (1.2)	19.7 (7.2)
	SDF	15	21.9 (0.8)	25.0 (12.2)	23.2 (0.8)	18.7 (6.4)
	RTT	14	22.6 (0.7)	26.3 (12.1)	22.4 (1.1)	21.0 (9.6)
Control	LDE	18	22.7 (0.5)	23.3 (8.5)	23.3 (0.3)	24.3 (5.0)
	SDF	17	22.7 (0.4)	23.0 (7.9)	23.0 (0.1)	25.3 (8.0)
	RTT	18	22.5 (0.3)	23.7 (10.3)	23.7 (0.4)	23.3 (8.0)
Hydration	LDE	15	22.3 (1.0)	22.7 (8.0)	–	–
	SDF	15	22.3 (1.2)	22.7 (7.6)	–	–
	RTT	13	22.3 (1.0)	22.7 (13.8)	–	–

Data presented as mean (SD). For the Hydration study, post imaging occurred in solution. LDE and SDF: lateral digital extensor and superficial digital flexor tendons from bovine forelimb; RTT is rat tail tendon.

### Data Analysis

**Fig. 7:**
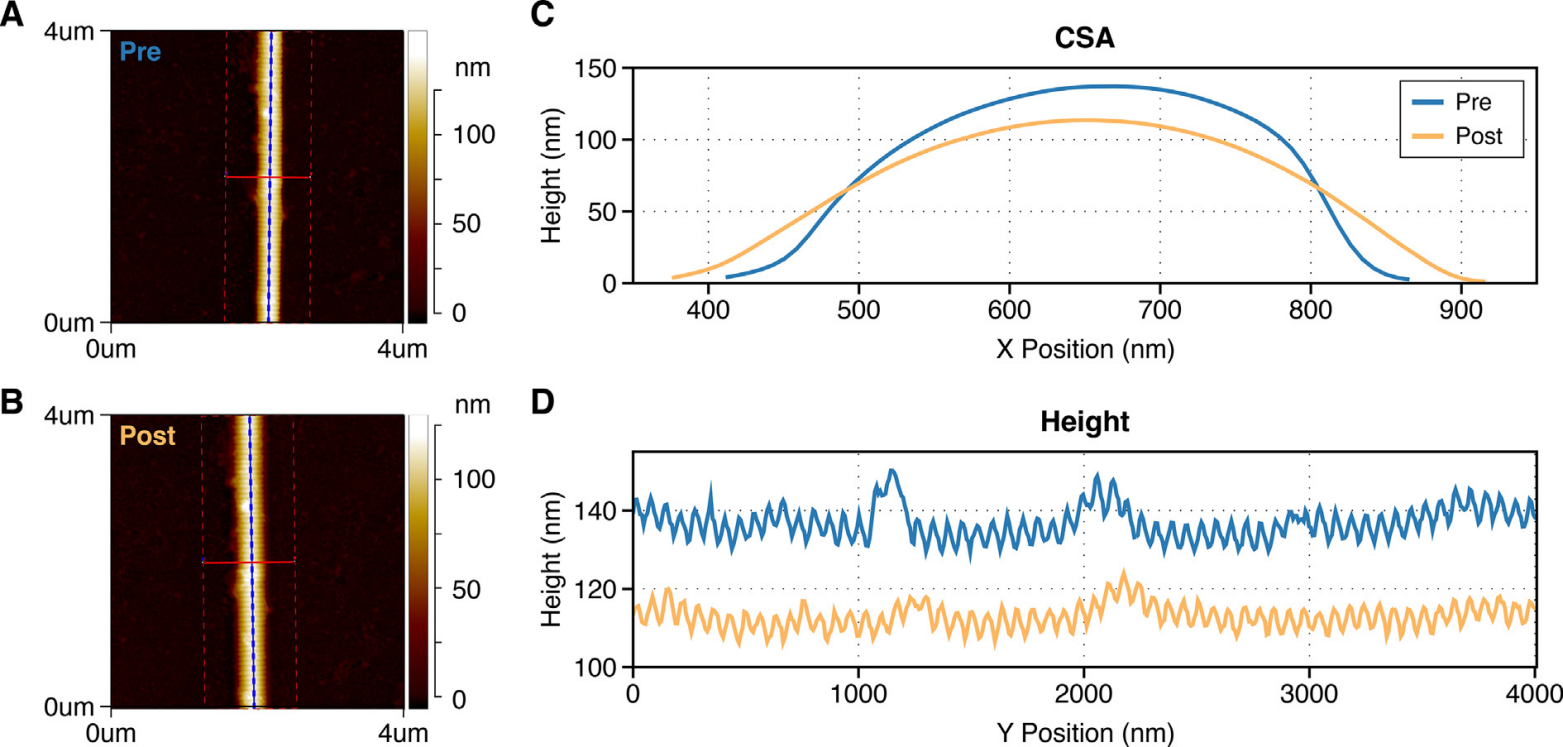
AFM images of the same 4 μm length of the same rat tail tendon collagen fibril imaged before **(A)** and after **(B)** exposure to MMP-1, with corresponding pre- and post-treatment cross-section **(C)** and longitudinal height profiles **(D)**.

AFM images were analyzed to obtain an average height and cross-section profile of each fibril studied. Images were processed using Nanoscope Analysis (v1.4, Bruker, USA) and Scanning Probe Image Processor (SPIP™) software (v6.3.0, Image Metrology, Denmark), with zero force height calculated by addition of height and deformation channels. An average height profile was created by extracting a 5-pixel-wide line along the apex of each fibril, while cross-sections were taken through each line along the length of the fibrils to create an average cross-section. (An example of this can be seen in Fig. 7.) Height and cross-section profiles were analyzed with a custom MATLAB (vR2021b, MathWorks, USA) code to calculate average height, D-band length (determined using a Fast-Fourier transform of the height profile), cross-sectional area (CSA) (measured via integration of the average cross-section profile), and aspect ratio (AR) (width at half maximum height divided by height). For each fibril these structural measurements were made both before and after the treatment for each study (MMP-1 exposure for enzyme study, and MMP Buffer exposure only for control and hydration studies). Change in CSA and AR following treatment (post minus pre) was calculated. (An example of each fibril type can be seen in Fig. S2.) For fibrils imaged in the hydration study, hydration ratio was also calculated for CSA as well as height and width (at half maximum height) by dividing the hydrated dimension by the dry dimension. (An example of hydrated images of each fibril type can be seen in Fig. S1).

### Statistical methods

Statistical tests were performed using JMP software (v16.2, SAS Institute, USA). Figures containing box plots indicate the median (–), mean (X), interquartile range (length of box), and min/max values (whiskers).

**Fig. 8:**
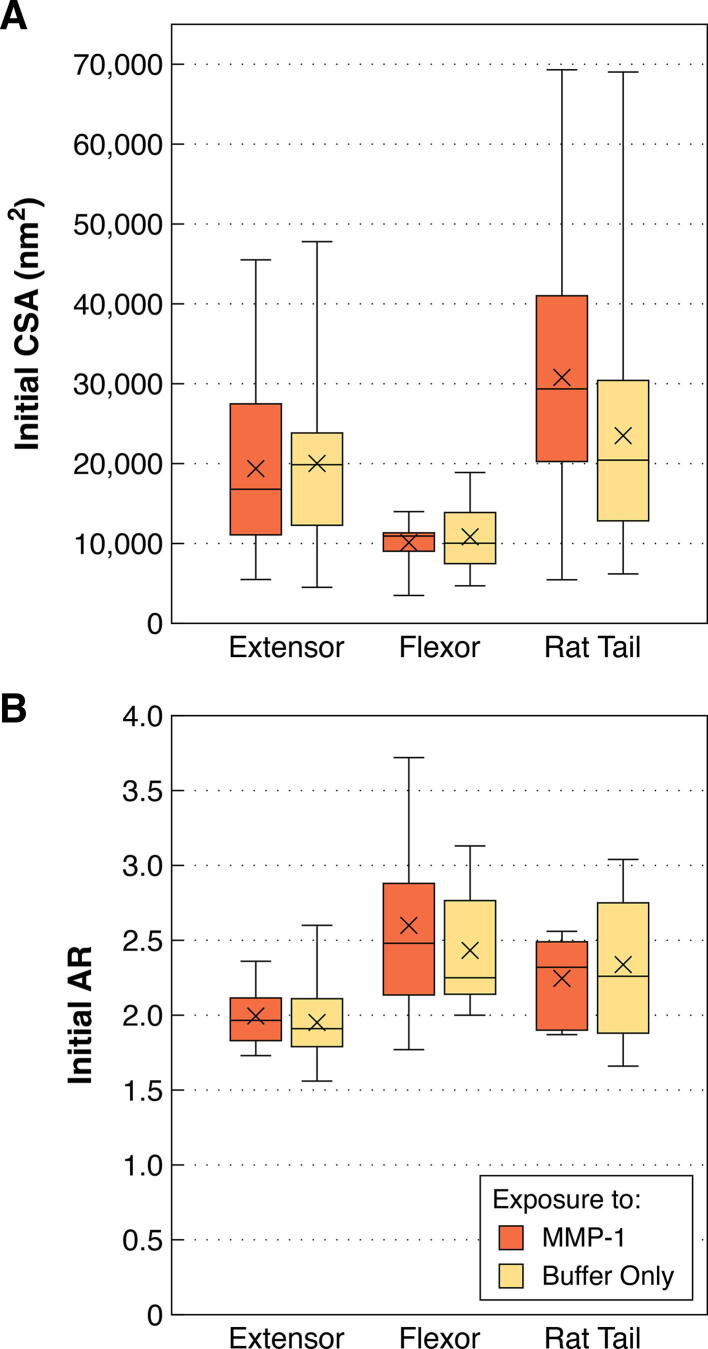
**A)** Cross-sectional area (CSA) and **B)** aspect ratio (AR) of individual collagen fibrils isolated from bovine extensor, bovine flexor, and rat tail tendons prior to treatment with MMP-1 in buffer (enzyme) or buffer alone (control). Within each tendon type, the fibrils belonging to the two study groups were equivalent prior to treatment.

The initial size (CSA) and shape (AR) of collagen fibrils sampled in the enzyme and control studies were compared within each tendon type using *t*-tests to confirm similarity of populations (Fig. 8). For LDE and RTT CSA, and RTT and SDF AR comparisons, non-parametric Wilcoxon tests were required. Because there were no significant differences between the fibrils used in these studies for either measure, the data were pooled across studies and a non-parametric 1-way ANOVA (Kruskal-Wallis) was used to compare between tendon types, with post-hoc multiple comparisons made using the Dunn method for joint ranking.

Change in CSA and AR following treatment for enzyme and control studies were compared within each tendon type using Welch’s test for LDE and RTT CSA and LDE AR comparisons and non-parametric Wilcoxon test for SDF CSA and AR and RTT AR comparisons.

Due to the significantly smaller size of the bovine flexor fibrils (Fig. 8), a subsample of comparably sized extensor fibrils with CSAs less than 15,000 nm^2^ was separately compared to the flexor fibrils in a similar manner. This included eight extensor fibrils from the enzyme study and nine from the control study. Initial CSA and change in CSA between treatments were compared between extensor fibrils smaller than 15,000 nm^2^ and all flexor fibrils using *t*-tests. No similar comparison using RTT fibrils could be done, as there were only two RTT fibrils with CSA smaller than 15,000 nm^2^.

To examine for an effect of fibril size on degradation, LDE and RTT samples were examined for relationships between initial CSA and change in CSA following treatment using linear regressions. This was done first for control fibrils, with the resulting linear relationship (or mean for insignificant relationship) used to subtract the correctly scaled CSA change for control samples from the CSA change for enzyme treated samples, thus giving the relative CSA change between enzyme and control groups: CSA (Enz-Con). Relationships between initial CSA and change in CSA due to MMP-1 exposure (CSA (Enz-Con)) were compared between tendons using an ANCOVA. The relationship between initial AR and change in AR with treatment was also examined using linear regressions for each tendon.

Hydration ratios were compared between tendon types for each measure (CSA, height, and width) using a 1-way ANOVA, with multiple comparisons made using a Holm-Bonferroni post-hoc adjustment. Dry and wet aspect ratios and the change in AR from dry to wet states were compared between tendons using a Welch’s 1-way ANOVA with multiple comparisons made using a Games-Howell post-hoc adjustment (to account for unequal variance).

## CRediT authorship contribution statement

**Kelsey Y. Gsell:** Conceptualization, Methodology, Software, Validation, Formal analysis, Investigation, Writing – original draft, Writing – review & editing, Visualization. **Samuel P. Veres:** Conceptualization, Formal analysis, Resources, Writing – review & editing, Supervision, Project administration, Funding acquisition. **Laurent Kreplak:** Conceptualization, Resources, Writing – review & editing, Supervision, Project administration, Funding acquisition.

## Declaration of Competing Interest

The authors declare that they have no known competing financial interests or personal relationships that could have appeared to influence the work reported in this paper.

## Data Availability

Data will be made available on request.
