# Supplementary material for: Single collagen fibrils isolated from high stress and low stress tendons show differing susceptibility to enzymatic degradation by the interstitial collagenase matrix metalloproteinase-1 (MMP-1)
Source: Matrix Biol Plus. 2023 Feb 21;18:100129. doi: 10.1016/j.mbplus.2023.100129 (PMC10006499; doi:10.1016/j.mbplus.2023.100129)
Supplement: Supplementary data 1 [file mmc1.docx]

**SUPPLEMENTARY MATERIAL**

**Single collagen fibrils isolated from high stress and low stress tendons show differing susceptibility to enzymatic degradation by the interstitial collagenase matrix metalloproteinase-1 (MMP-1)**

Kelsey Y. Gsell^a^, Samuel P. Veres^a,b,*^, Laurent Kreplak^a,c,*^

^a^ School of Biomedical Engineering, Dalhousie University, Halifax, Nova Scotia, Canada

^b^ Division of Engineering, Saint Mary’s University, Halifax, Nova Scotia, Canada

^c^ Physics and Atmospheric Science, Dalhousie University, Halifax, Nova Scotia, Canada

*Corresponding authors.

Email addresses: [kreplak@dal.ca](mailto:kreplak@dal.ca) (L Kreplak); [sam.veres@smu.ca](mailto:sam.veres@smu.ca) (SP Veres)

**
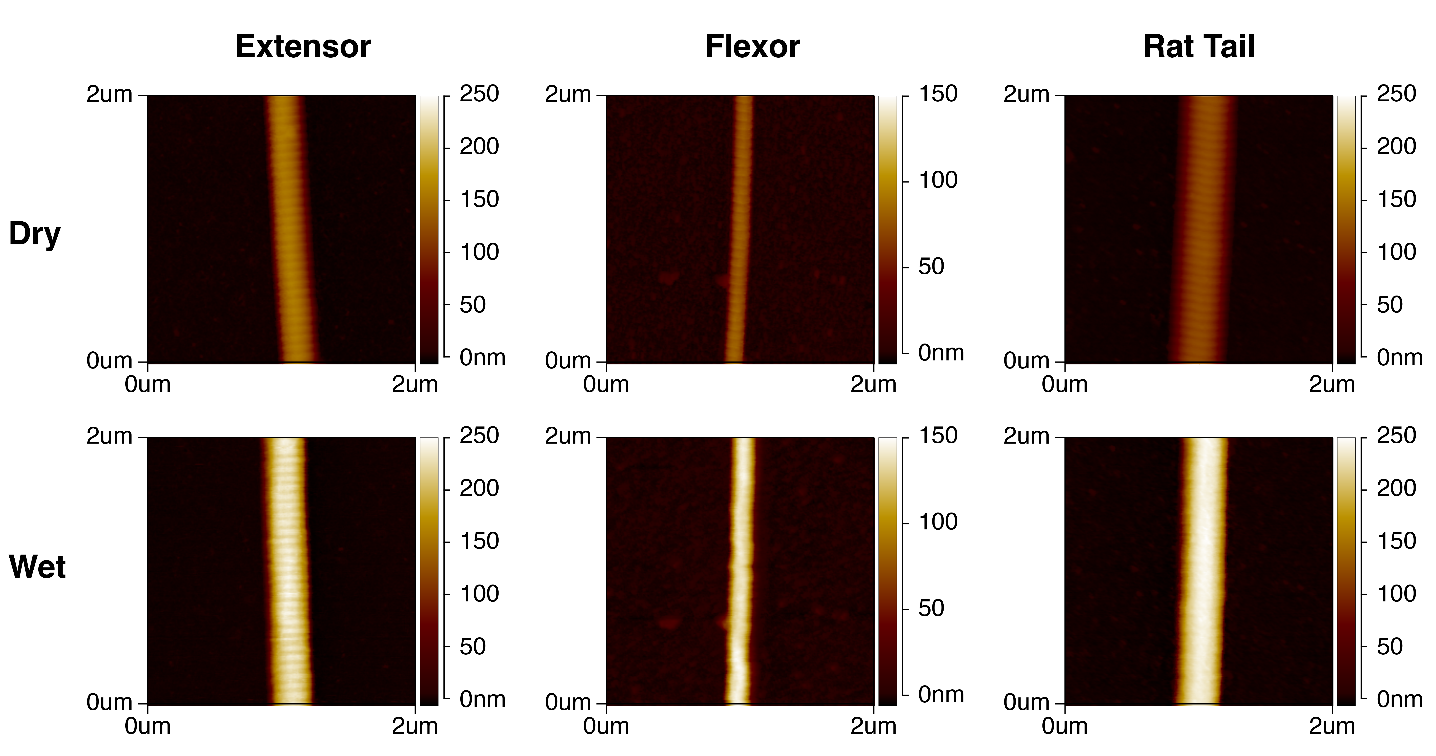
**

**Fig. S1.** Representative AFM images of a collagen fibril from paired bovine superficial digital flexor and lateral digital extensor tendons and rat tail tendon. The same segment of each fibril is imaged before (dry) and after (wet) hydration in MMP buffer. Height on the Z-axis is calculated as zero-force height (height + deformation).

**
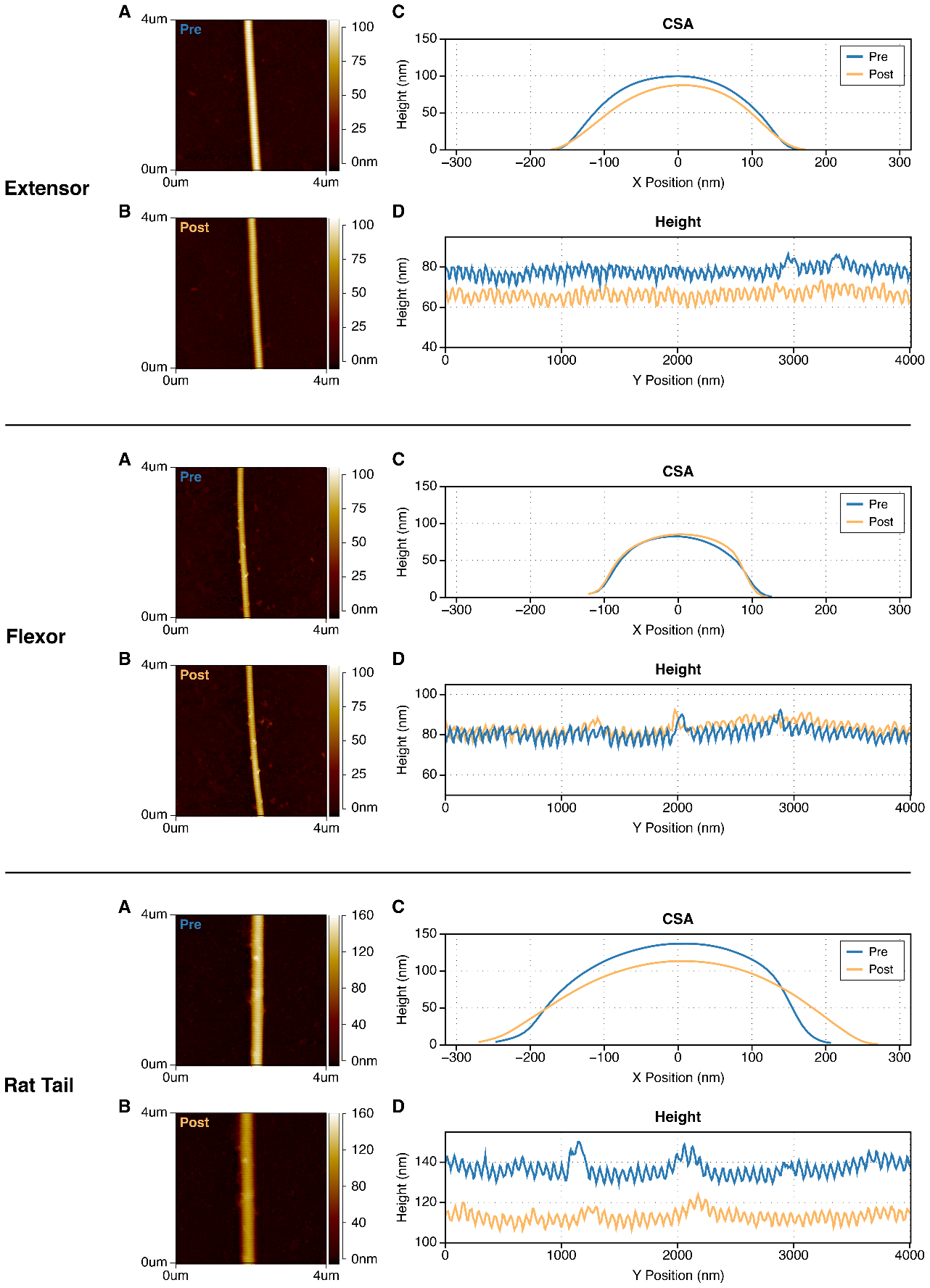
**

**Fig. S2.** Representative AFM images of a collagen fibril from paired bovine superficial digital flexor and lateral digital extensor tendons and rat tail tendon. The same 4 μm segment of each fibril is imaged dry before **(A)** and after **(B)** exposure to MMP-1, with corresponding pre- and post-treatment cross-section **(C)** and longitudinal height profiles **(D)**.
